# Sweet Potato Symptomless Virus 1: First Detection in Europe and Generation of an Infectious Clone

**DOI:** 10.3390/microorganisms10091736

**Published:** 2022-08-28

**Authors:** Elvira Fiallo-Olivé, Ana Cristina García-Merenciano, Jesús Navas-Castillo

**Affiliations:** Instituto de Hortofruticultura Subtropical y Mediterránea “La Mayora” (IHSM-UMA-CSIC), Consejo Superior de Investigaciones Científicas, Avenida Dr. Wienberg s/n, 29750 Algarrobo-Costa, Málaga, Spain

**Keywords:** sweet potato, mastrevirus, *Geminiviridae*, infectious clone, agroinoculation

## Abstract

Sweet potato (*Ipomoea batatas*), a staple food for people in many of the least developed countries, is affected by many viral diseases. In 2017, complete genome sequences of sweet potato symptomless virus 1 (SPSMV-1, genus *Mastrevirus*, family *Geminiviridae*) isolates were reported, although a partial SPSMV-1 genome sequence had previously been identified by deep sequencing. To assess the presence of this virus in Spain, sweet potato leaf samples collected in Málaga (southern continental Spain) and the Spanish Canary Islands of Tenerife and Gran Canaria were analyzed. SPSMV-1 was detected in samples from all the geographical areas studied, as well as in plants of several entries obtained from a germplasm collection supposed to be virus-free. Sequence analysis of full-length genomes of isolates from Spain showed novel molecular features, i.e., a novel nonanucleotide in the intergenic region, TCTTATTAC, and a 24-nucleotide deletion in the V2 open reading frame. Additionally, an agroinfectious clone was developed and infectivity assays showed that the virus was able to asymptomatically infect *Nicotiana benthamiana*, *Ipomoea nil*, *I*. *setosa*, and sweet potato, thus confirming previous suggestions derived from observational studies. To our knowledge, this is the first report of the presence of SPSMV-1 in Spain and Europe and the first agroinfectious clone developed for this virus.

## 1. Introduction

Sweet potato (*Ipomoea batatas*) is one of the most consumed crops in the world and is a staple food for people in many of the least developed countries. This crop is the sixth most important food crop worldwide after rice, wheat, potato, maize, and cassava. Sweet potato crops produce more edible energy per hectare per day than other important crops, such as wheat, rice, and cassava, and are an important source of carbohydrates, beta-carotene, and vitamins B, C, and E. Sweet potato is vegetatively propagated by vine cuttings, which cause the accumulation of pathogens, mainly viruses. Consequently, this crop is particularly affected by viral diseases that substantially decrease the quality and yield of the storage roots [1].

In Europe, the demand for sweet potatoes has greatly increased in recent years, leading to an increase in imports and production in southern European countries, including Spain. Although extensive field studies of the losses caused by sweet potato viruses have not been carried out in Spain, several viruses have been reported to infect this crop. They are the crinivirus (genus *Crinivirus*, family *Closteroviridae*) sweet potato chlorotic stunt virus [2]; the potyviruses (genus *Potyvirus*, family *Potyviridae*) sweet potato feathery mottle virus, sweet potato virus G, and sweet potato virus 2 [2,3]; and the begomoviruses (genus *Begomovirus*, family *Geminiviridae*) sweet potato leaf curl virus and sweet potato leaf curl Canary virus [4,5]. In addition, the presence of deltasatellites (genus *Deltasatellite*, family *Tolecusatellitidae*) associated with the begomoviruses infecting sweet potato has also been reported in this country [6,7].

The genus *Mastrevirus* is one of 14 genera in the family *Geminiviridae* [8]. Mastreviruses infect dicot and monocot plant species and are transmitted by leafhoppers. Like all members of the family *Geminiviridae*, mastrevirus genomes are encapsidated in unique twinned (geminate) icosahedral particles [8]. Mastrevirus genomes have a single component of circular single-stranded DNA of 2.6–2.8 kb containing four open reading frames (ORFs) encoded on the virion-sense (V1, coat protein and V2, movement protein) or the complementary-sense strands (C1 and C1:C2, proteins involved in replication). An intron occurs between ORFs C1 and C2 of all mastrevirus genomes. A short and a long intergenic region are also present; the latter contains sequence elements necessary for viral replication and transcription, including a stem-loop motif containing the canonical nonanucleotide sequence TAATATTAC [8].

In 2009, the partial genome sequence of a mastrevirus was identified in sweet potato by the deep sequencing of small RNAs in Peru [9]. However, it was not until 2017 that the complete genome sequence of the virus was obtained [10]. Until now, the presence of the virus, named sweet potato symptomless virus 1 (SPSMV-1), has been reported to infect sweet potato plants from Brazil, China, Ecuador (Galapagos Islands), Kenya, Korea, Peru, Taiwan, Tanzania, Uruguay, and the USA [10,11,12,13].

In this study, the presence of SPSMV-1 was assessed for the first time in Spain, with the virus found in samples from all of the geographical areas studied. This is the first record of the virus in Europe. SPSMV-1 was also detected in several samples of pathogen-tested in vitro plants obtained from a germplasm collection. In addition, the first agroinfectious clone of the virus was developed, and infectivity assays showed that it was able to infect *Nicotiana benthamiana*, *Ipomoea nil*, *I*. *setosa,* and sweet potato with no noticeable symptoms.

## 2. Materials and Methods

### 2.1. Plant Samples

Leaf samples were collected from 95 plants from Málaga province (southern continental Spain) and the Spanish Canary Islands of Tenerife and Gran Canaria (Table 1 and Table S1). Sampled plants included cultivated sweet potato (*I*. *batatas*) (*n* = 71) from Málaga, Tenerife, and Gran Canaria and ornamental or naturalized *I*. *indica* (*n* = 24) from Tenerife and Gran Canaria. Most of the plants did not show any conspicuous symptoms.

### 2.2. DNA Extraction, PCR Amplification, and Cloning

Total DNA was extracted from leaf samples using a cetyltrimethylammonium bromide (CTAB)-based purification method [14]. PCR to detect sweet potato symptomless virus 1 was carried out using primers Detect-1F (5′-CCTAAGTCGTCGTCCGATAG-3′)/Detect-1R (5′-TTGAGTCCAGGTAAACTGAGC-3′) and Full-4F (5′-TGGATATTAGTAAACCGGGTCA-3′)/Full-4R (5′-CACCATTCGACGTCACAA-3′) [10]. PCR was carried out with BIOTAQ DNA polymerase (Bioline, London, UK). For primers Detect-1F/Detect-1R, the first PCR step was denaturation for 3 min at 95 °C, followed by 34 cycles of denaturation for 45 s at 95 °C, hybridization for 45 s at 52 °C, and extension for 45 s at 72 °C, followed by a final extension step of 5 min at 72 °C. For primers Full-4F/Full-4R, a denaturation for 3 min at 95 °C was used, followed by 34 cycles of denaturation for 1 min at 95 °C, hybridization for 1 min at 53 °C, and extension for 3 min at 72 °C, followed by a final extension step of 5 min at 72 °C. In addition, nested PCR was designed to amplify 329 nt of the coat protein gene of the virus. The first PCR was carried out with primers MA2924 (5′-CTACCTGGGATGATGTGGCTAGAC-3′)/MA2925 (5′-CCATTCGACGTCACAATCGTCTTC-3′) (first denaturation step of 95 °C for 3 min, followed by 34 cycles of 30 s at 95 °C, 30 s at 61.7 °C, and 30 s at 72 °C, and a final step of 5 min at 72 °C). The second PCR was carried out with primers MA2926 (5′-CTACGAGATCGACCGAGTCTGCAG-3′)/MA2927 (5′-GCAACAGTCCACGTATTTGGGAAG-3′) (first denaturation step of 95 °C for 3 min, followed by 34 cycles of 30 s at 95 °C, 30 s at 64.2 °C, and 30 s at 72 °C, and a final step of 5 min at 72 °C). DNA fragments obtained with primers Detect-1F/Detect-1R and MA2926/MA2927 were directly sequenced. DNA fragments obtained with Full-4F/Full-4R and MA2782/MA2784 were cloned into the pGEM-T-Easy Vector (Promega, Madison, WI, USA), and inserts of selected clones were sequenced. All sequence reactions were carried out at Macrogen Inc. (Seoul, Korea).

### 2.3. Sequence Analysis

Sequences were analyzed with the Lasergene sequence analysis package (DNAStar Inc., Madison, WI, USA). The BLASTn algorithm was used to determine sequence similarity in the GenBank database. The BLASTn program [15] (http://www.ncbi.nlm.nih.gov/Blast.cgi (accessed on 1 May 2022)) was used to perform the sequence similarity search. Pairwise identity scores were calculated with the Sequence Demarcation Tool (SDT) [16] after sequence alignment with MUSCLE [17]. The best-fit model of nucleotide substitution was determined based on the corrected Akaike and Bayesian information criteria, as implemented in MEGA7 [18].

### 2.4. Development of an Agroinfectious Clone and Plant Agroinoculation

Primers MA2782 (GGTACCGTGTATTTGATGACGATGTAC)/MA27784 (GGTACCCCCTGGGTTGAACACAAC) were designed to amplify the full-length genome of an isolate of SPSMV-1 present in sample B2 from Málaga (GenBank accession numbers ON526997). These primers included the sequence of the *Kpn*I restriction site (underlined) naturally present in the viral genome sequence. PCR was carried out as follows: denaturation for 3 min at 95 °C, 34 cycles of denaturation for 1 min at 95 °C, hybridization for 1 min at 66 °C, and extension for 3 min at 72 °C, followed by a final extension step of 5 min at 72 °C. The infectious dimeric SPSMV-1 clone was constructed essentially as described by Ferreira et al. [19]. The full-length SPSMV-1 genome was released with *Kpn*I from the pGEM-T-Easy Vector, religated, and used as a template for rolling circle amplification (RCA) (TempliPhi DNA Amplification Kit, GE Healthcare, Little Chalfont, UK). The RCA product was partially digested with *Kpn*I to produce dimeric molecules that were cloned in the pUC18 vector. The insert of the dimeric clone was excised with *Bam*HI/*Eco*53KI and subcloned in the *Bam*HI/*Sma*I sites of the pCAMBIA0380 binary vector.

An *Agrobacterium tumefaciens* C58C1 culture harboring the dimeric clone of SPSMV-1 was grown for 2 days at 28 °C in yeast extract peptone (YEP) liquid medium containing kanamycin (50 µg/mL) and rifampicin (50 µg/mL). For agroinoculation assays, *A*. *tumefaciens* cultures were used to inoculate plants by stem puncture, as previously described [20,21]. Plants inoculated with *A*. *tumefaciens* C58C1 cultures containing the empty pCAMBIA0380 vector were used as negative controls (mock). *Nicotiana benthamiana* was inoculated at the four-leaf stage. *Ipomoea nil* and *I*. *setosa* were inoculated at the two-leaf stage and cuttings of sweet potato cv. ‘Tanzania’ and ‘Camote Morado’ were inoculated. Two independent experiments were carried out for each plant species. Plants were maintained in an insect-free growth chamber (25/18 °C day/night, 70% relative humidity, and a 16 h photoperiod at 250 µmol s^−1^ m^−2^ of photosynthetically active radiation) until analyzed.

## 3. Results

### 3.1. Sweet Potato Symptomless Virus 1 Naturally Infects Sweet Potato in Continental Spain and the Canary Islands

**Figure 1:**
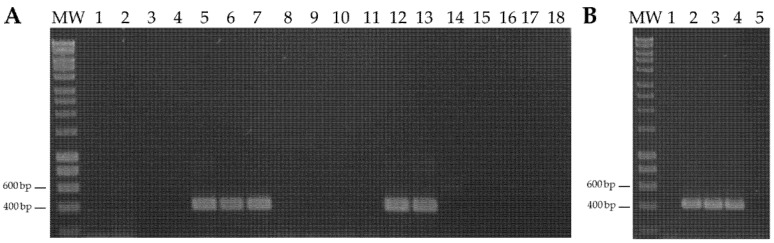
Agarose gel electrophoresis of PCR products of sweet potato symptomless virus 1 using primers Detect-1F/1R. MW, HyperLadder 1kb (Bioline). (**A**) Lanes 1–18, sweet potato samples CI62–CI79 from the Canary Islands. (**B**) Samples from a sweet potato germplasm collection. Lanes 1–5, cv. ‘Tanzania,’ ‘Blanca,’ ‘Beauregard,’ ‘Promesa’, and ‘Camote Morado,’ respectively.

The DNA extracted from the leaf samples of sweet potato and *I. indica* plants from Málaga (southern continental Spain) and the Spanish Canary Islands of Tenerife and Gran Canaria was analyzed for the presence of SPSMV-1. PCR with primers Detect-1F/Detect-1R showed the expected DNA fragment of 418 bp in sweet potato samples from all locations (Tenerife, Gran Canaria, and Málaga) (Table 1 and Table S1; Figure 1A). Overall, 36 of 71 sweet potato samples were infected with SPSMV-1 (16/34 from Tenerife, 14/22 from Gran Canaria, and 6/15 from Málaga). In contrast, none of the *I. indica* samples showed the expected DNA fragments (Table 1 and Table S1).

### 3.2. Sweet Potato Symptomless Virus 1 Is Present in Pathogen-Tested In Vitro Plants Obtained from a Germplasm Collection

Sweet potato plants of cultivars ‘Tanzania’, ‘Blanca,’ ‘Beauregard,’ ‘Promesa’, and ‘Camote Morado’ obtained from the USDA-ARS Plant Genetic Resources Conservation Unit (Griffin, GA, USA) and maintained by cuttings in insect-proof chambers for more than 10 years were screened with primers Detect-1F/Detect-1R to detect SPSMV-1. Surprisingly, the virus was detected in cultivars ‘Blanca’, ‘Beauregard’, and ‘Promesa’ (Figure 1B). To ensure that the virus was not present in sweet potato cultivars ‘Tanzania’ and ‘Camote Morado’ thus allowing the use of these cultivars in agroinoculation experiments, 10 leaf samples of each cultivar were used to individually detect the presence of the virus using nested PCR with primers MA2924/MA2925 and MA2926/MA2927; none of the 20 samples amplified the expected DNA fragment of 329 nt (Figure S1).

### 3.3. Sweet Potato Symptomless Virus 1 Isolates from the Canary Islands and Málaga Are Closely Related with Isolates from All over the World but Contain Novel Molecular Features

To molecularly characterize SPSMV-1 isolates from Spain and establish the phylogenetic relationships with isolates from other countries, the full-length viral genome was amplified with primers Full-4F/Full-4R from samples CI24 from Tenerife; CI61 from Gran Canaria; and B2, B3, and B14 from Málaga. The expected DNA fragments corresponding to the full-length SPSMV-1 genomes were cloned into the pGEM-T-Easy Vector. Two clones were sequenced per sample and deposited in the GenBank database under accession numbers ON526993–ON527002.

**Figure 2:**
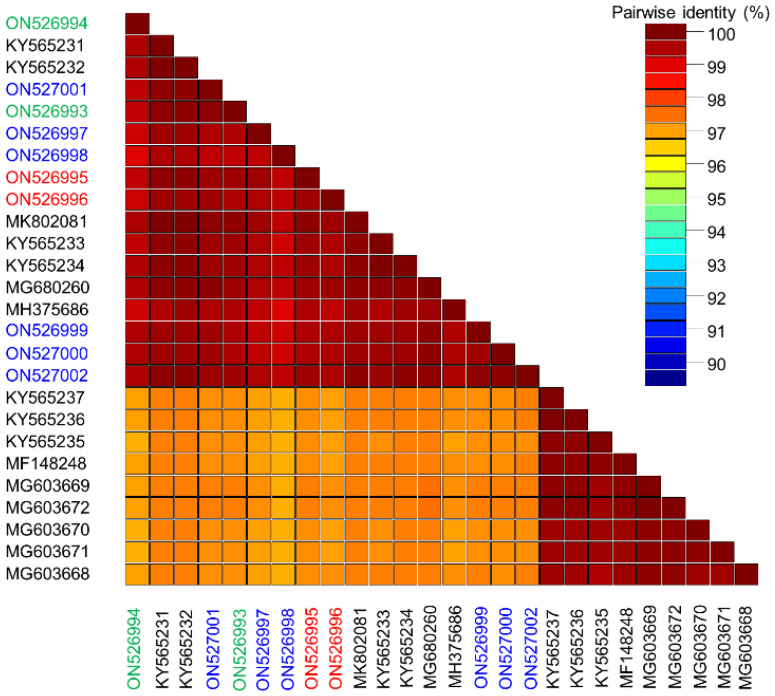
Color-coded matrix of pairwise sequence identity scores generated by the alignment of the full-length genomes of sweet potato symptomless virus 1 (SPSMV-1) obtained in this work. Samples from the Canary Islands of Tenerife and Gran Canaria are in green and red, respectively, and those from Málaga are in blue. All other full-length genomes of SPSMV-1 available from GenBank (in black) are included in the analysis.

Sequences obtained in this work showed a similarity of 99.0–99.8% and at least 96.9% similarity with all SPSMV-1 isolates available in GenBank (Figure 2). The unusual nonanucleotide TAAGATTCC present in most Spanish SPSMV-1 sequences (9 out of 10, (ON526993–ON526997, ON526999–ON527002)) is coincident with the nonanucleotide previously reported for the virus [10]. However, one of the clones obtained from sample B2 contained a different nonanucleotide sequence, TCTTATTAC (ON526998). The full-length SPSMV-1 sequences obtained in this work ranged from 2578 to 2602 nt. This difference in size occurred because several isolates (ON526994, ON526999, ON527000, and ON527002) showed a deletion of 24 nucleotides near the 5*′* end of V2 ORF. The movement protein encoded by the V2 ORF in the mentioned isolates lacked the corresponding eight amino acids present in all previously sequenced SPSMV-1 isolates deposited in GenBank.

**Figure 3:**
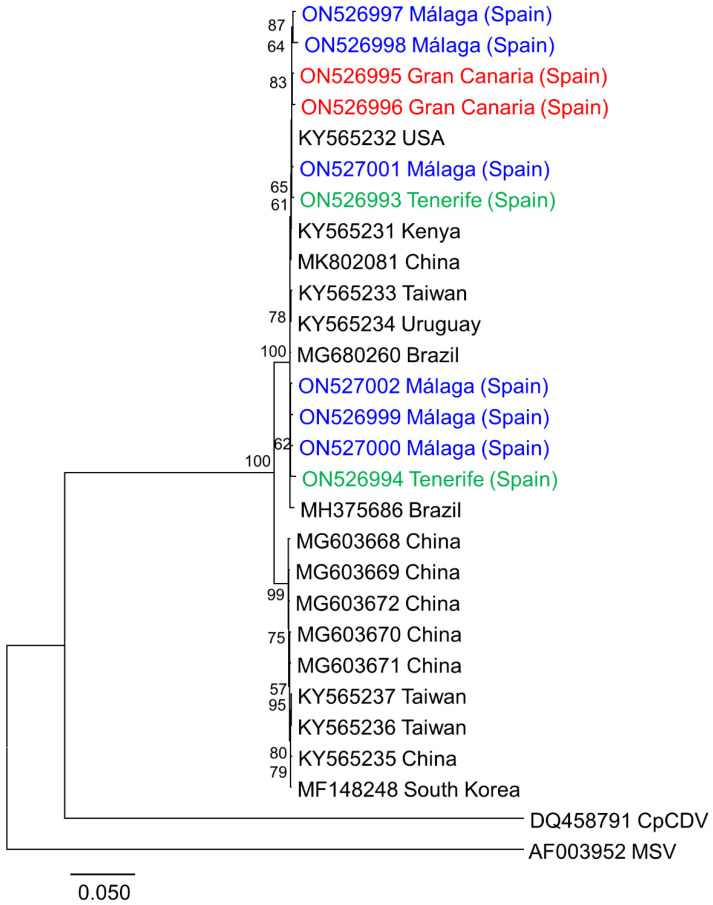
Phylogenetic tree illustrating the relationships of the sweet potato symptomless virus 1 (SPSMV-1) genomes obtained in this work with SPSMV-1 isolates previously reported and one representative isolate each of the dicot-infecting mastrevirus chickpea chlorotic dwarf virus (CpCDV) and the monocot-infecting mastrevirus maize streak virus (MSV). Samples were obtained from Málaga (southern continental Spain) (blue) and the Canary Islands of Tenerife (green) and Gran Canaria (red). The tree was constructed using the maximum likelihood method with the MEGA 7 program using the best fit model. HKY and bootstrap values (1000 replicates) are shown for supported branches (>50%). The bar below the tree indicates 0.050 nucleotide substitutions per site.

Phylogenetic analysis of all SPSMV-1 isolates available, including those obtained in this work, showed no correlation between the isolates and their geographic origin (Figure 3).

### 3.4. A Dimeric Sweet Potato Symptomless Virus 1 Clone Is Infectious in N. benthamiana, I. nil, I. setosa, and Sweet Potato

**Figure 4:**
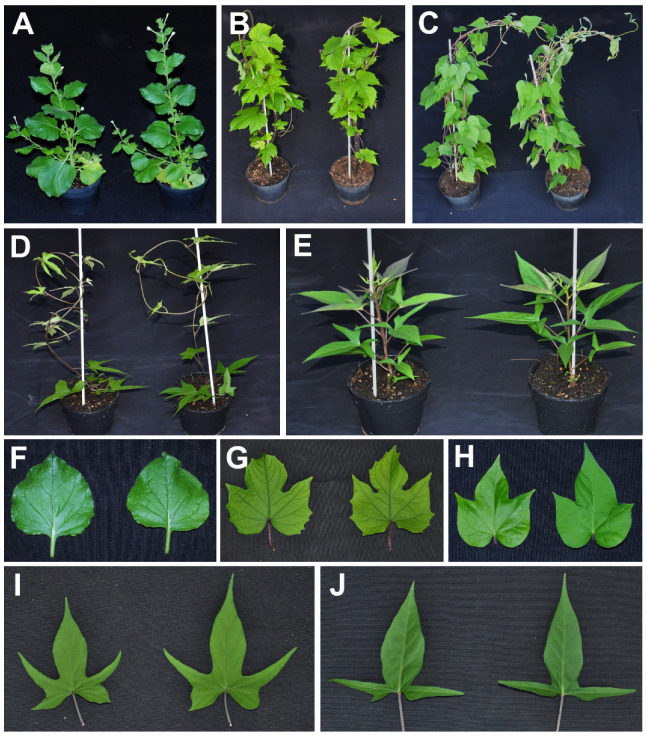
Plant and leaf details of *Nicotiana benthamiana* (**A**,**F**), *Ipomoea setosa* (**B**,**G**), *Ipomoea nil* (**C**,**H**), sweet potato cv. ‘Tanzania’ (**D**,**I**) and ‘Camote Morado’ (**E**,**J**) infected with the sweet potato symptomless virus 1 infectious clone. In each panel, mock-inoculated plants are shown on the left and infected plants on the right. Photographs of representative plants were taken 40 days post-inoculation.

A dimeric clone constructed for an SPSMV-1 isolate from Málaga was used to agroinoculate a range of plant species; the experimental hosts *N. benthamiana*, *I. nil,* and *I. setosa*; and the natural host sweet potato (cv. ‘Tanzania’ and ‘Camote Morado’). Plants inoculated in two independent experiments and analyzed by nested PCR with primers MA2924/MA2925 and MA2926/MA2927 at 5 weeks post-inoculation showed that the virus was able to infect all of the plant species assayed (Table 2, Figure S2). The proportion of infected plants ranged from 12.5% for *I. nil* to 45.83% for *N. benthamiana*. No evident symptoms were observed in any of the inoculated plants, suggesting that the Spanish SPSMV-1 isolate does not cause symptoms in these hosts (Figure 4).

## 4. Discussion

Mastrevirus SPSMV-1 has been reported to infect sweet potato plants in several countries around the world [10,11,12,13]. In this work, the presence of the virus was assessed for the first time in Spain. SPSMV-1 was detected in approximately half of the sweet potato plants analyzed (50.70%) and was present in all sampled areas, Málaga (40%) in southern continental Spain and the Spanish Canary Islands of Tenerife (47.05%) and Gran Canaria (63.63%). To our knowledge, this is the first report of the presence of SPSMV-1 in Spain and Europe. Unexpectedly, the virus was also found infecting three sweet potato cultivars (‘Blanca’, ‘Beauregard’, and ‘Promesa’) that were supposed to be virus-free, as they were obtained as pathogen-tested in vitro plants maintained in a germplasm collection. This highlights the importance of developing methods to detect viruses present in germplasm collections that would otherwise remain undetected.

Sequence analysis of the full-length genomes of the SPSMV-1 isolates reported here showed a high sequence identity and a close phylogenetic relationship between them and the isolates available from GenBank. As with all SPSMV-1 isolates previously characterized [10], most of the isolates sequenced in this work showed the presence of the unusual nonanucleotide TAAGATTCC. However, a unique novel nonanucleotide, TCTTATTAC, was found in an isolate from Málaga. Another singularity of some of the SPSMV-1 isolates reported here is the presence of a shorter-than-usual V2 ORF with a 24-nt deletion close to the 5′ end, thus encoding a shorter putative movement protein. Interestingly, these deletion mutants, not previously described, were found in both the Canary Island of Tenerife and in Málaga.

In this study, we developed for the first time an agroinfectious clone for SPSMV-1. Our results showed that the clone was able to infect the model plant *N*. *benthamiana*, two species used as bioindicators for sweet potato viruses, *I*. *nil* and *I. setosa*, and two cultivars of sweet potato (‘Tanzania’ and ‘Camote Morado’). No evident symptoms of the disease were observed in any of the infected plants. Thus, our experimental results support previous observations that SPSMV-1 could not be associated with any conspicuous symptoms in sweet potato plants [9,10]. However, although this virus does not seem to play an important pathological role, at least in the sweet potato cultivars assayed, it may have an effect in the presence of other viruses. The occurrence of synergistic interactions between viruses infecting sweet potatoes has been widely reported [22,23]. The presence of symptomless/latent viruses is not uncommon in crops and sweet potato is not an exception. Examples of asymptomatic viruses infecting sweet potatoes include, in addition to SPSMV-1, the potyvirus sweet potato latent virus and the badnavirus sweet potato pakakuy virus [22,24].

In summary, SPSMV-1 was confirmed as an asymptomatic mastrevirus infecting sweet potato crops in an increasing number of countries including Spain. Several biological aspects remain to be elucidated about this virus, including the possible interactions with other viruses in mixed infections and the mode of transmission. The putative insect vector is likely a leafhopper, similar to other mastreviruses [8].

## Figures and Tables

**Table 1:** Sweet potato and *Ipomoea indica* samples analyzed in this study. Additional details are given in Table S1.

Location	No. of Infected Sweet Potato Plants/Total No. of Sweet Potato Plants (Percentage)	No. of Infected *I. indica* Plants/Total No. of *I. indica* Plants
Tenerife (Canary Islands)	16/34 (47.05%)	0/12
Gran Canaria (Canary Islands)	14/22 (63.63%)	0/12
Málaga (southern continental Spain)	6/15 (40%)	-
Total	36/71 (50.70%)	0/12

**Table 2:** Infectivity of sweet potato symptomless virus 1 in *Nicotiana benthamiana*, *Ipomoea nil*, *I. setosa,* and sweet potato plants. Data from two agroinoculation experiments and mock-inoculated plants were included.

	No. of Infected Plants/No. of Agroinoculated Plants		
Plant Species	Experiment 1	Experiment 2	Total (%)
*Nicotiana benthamiana*	9/12 0/12 (mock)	2/12 0/12 (mock)	45.83
*Ipomoea nil*	3/24 0/12 (mock)	3/24 0/12 (mock)	12.5
*Ipomoea setosa*	6/24 0/12 (mock)	9/24 0/12 (mock)	31.25
Sweet potato cv. ‘Tanzania’	2/18 0/5 (mock)	4/10 0/3 (mock)	21.42
Sweet potato cv. ‘Camote Morado’	2/13 0/5 (mock)	2/14 0/3 (mock)	14.81

## Data Availability

Nucleotide sequences obtained in this study have been deposited in GenBank under accession numbers ON526993-ON527002.

## Supplementary Materials

The following supporting information can be downloaded at: https://www.mdpi.com/article/10.3390/microorganisms10091736/s1, Figure S1: Agarose gel electrophoresis of nested PCR products of sweet potato symptomless virus 1 using primers MA2924/MA2925 and MA2926/MA2927; Figure S2: Agarose gel electrophoresis of nested PCR products from plants agroinoculated with sweet potato symptomless virus 1 using primers MA2924/MA2925 and MA2926/MA2927; Table S1: Information on the sweet potato and *Ipomoea indica* samples used in this study.
