# Supplementary material for: Sweet Potato Symptomless Virus 1: First Detection in Europe and Generation of an Infectious Clone
Source: Microorganisms. 2022 Aug 28;10(9):1736. doi: 10.3390/microorganisms10091736 (PMC9504438; doi:10.3390/microorganisms10091736)
Supplement: Supplementary file 1 [file microorganisms-10-01736-s001.zip › Figure S1.pdf]

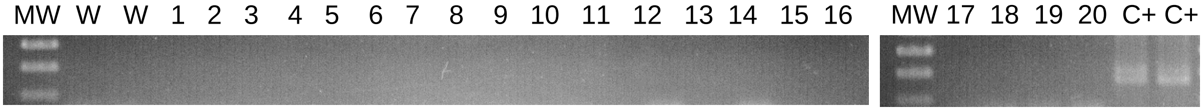

Figure S1. Agarose gel electrophoresis of nested PCR products of sweet potato symptomless virus 1 using primers MA2924/MA2925 and MA2926/MA2927. MW, HyperLadder 1kb (Bioline), W, water. Lanes 1–10, sweet potato cv. 'Camote Morado'; lanes 11–20, sweet potato cv. 'Tanzania.' C+, sweet potato cv. 'Blanca.'
