# Supplementary material for: Sweet Potato Symptomless Virus 1: First Detection in Europe and Generation of an Infectious Clone
Source: Microorganisms. 2022 Aug 28;10(9):1736. doi: 10.3390/microorganisms10091736 (PMC9504438; doi:10.3390/microorganisms10091736)
Supplement: Supplementary file 1 [file microorganisms-10-01736-s001.zip › Figure S2.pdf]

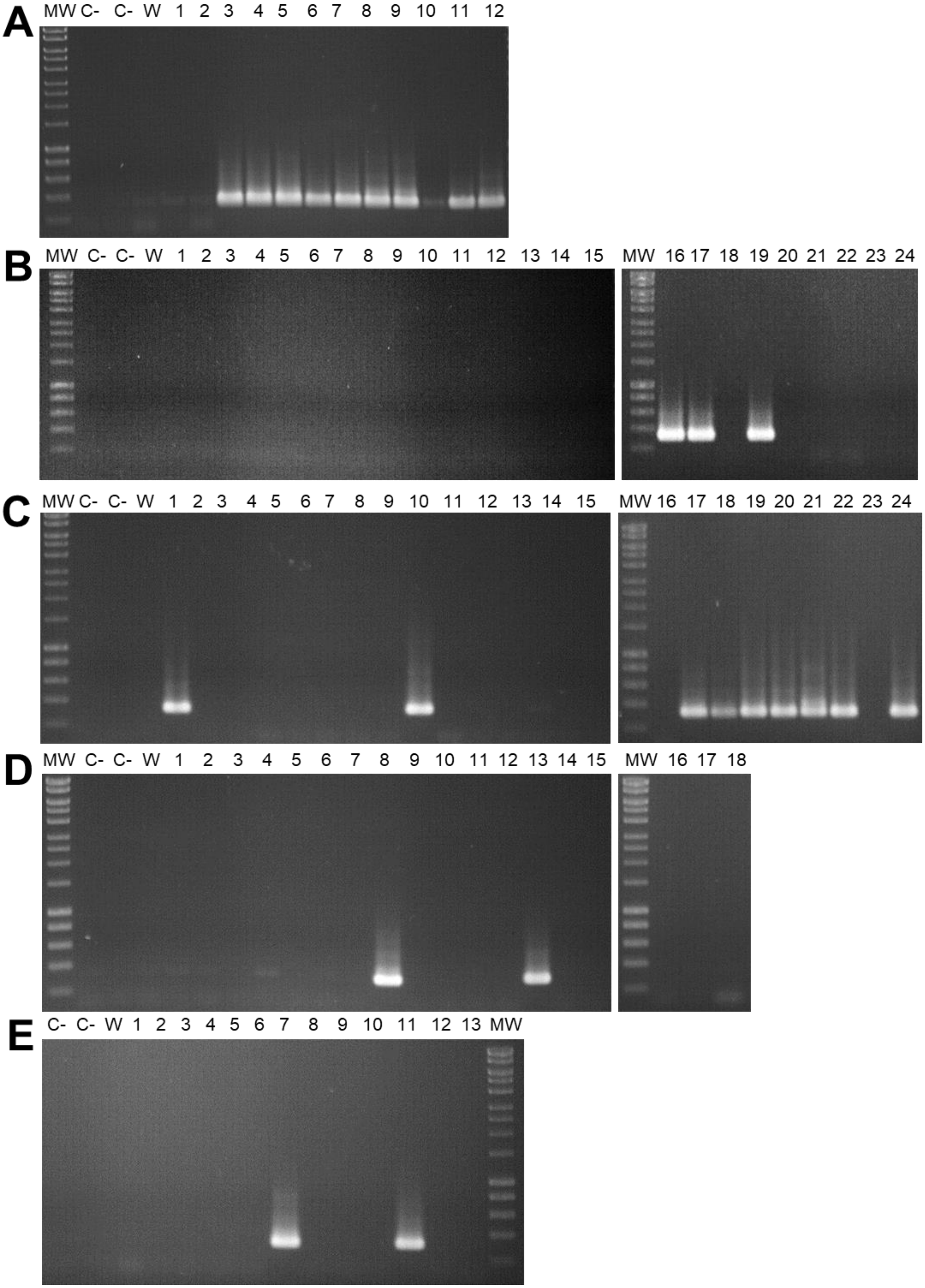

Figure S2. Agarose gel electrophoresis of nested PCR products from plants agroinoculated with sweet potato symptomless virus 1 using primers MA2924/MA2925 and MA2926/MA2927. MW, HyperLadder 1kb (Bioline); C-, mock inoculated plants; W, water. (A) *Nicotiana benthamiana*, (B) *Ipomoea nil*, (C) *I. setosa*, and (D) sweet potato cv. 'Tanzania' and (E) 'Camote Morado.'
