# Supplementary material for: Sweet Potato Symptomless Virus 1: First Detection in Europe and Generation of an Infectious Clone
Source: Microorganisms. 2022 Aug 28;10(9):1736. doi: 10.3390/microorganisms10091736 (PMC9504438; doi:10.3390/microorganisms10091736)
Supplement: Supplementary file 1 [file microorganisms-10-01736-s001.zip › Table S1.pdf]

Table S1. Information on the sweet potato and *Ipomoea indica* samples used in this study.

Sample code	Location	Coordinates	Host	Symptoms	SPSMV-1*
CI1	Tenerife	28°30.814'N 16°23.159'W	*Ipomoea indica*	No symptoms	-
CI2	Tenerife	28°30.824'N 16°23.154'W	*Ipomoea indica*	No symptoms	-
CI3	Tenerife	28°34.344'N 16°19.845'W	Sweet potato	No symptoms	-
CI4	Tenerife	28°34.345'N 16°19.853'W	Sweet potato	No symptoms	-
CI5	Tenerife	28°34.355'N 16°19.843'W	Sweet potato	No symptoms	-
CI6	Tenerife	28°34.356'N 16°19.843'W	Sweet potato cv. Patagallo	No symptoms	+
CI7	Tenerife	28°34.346'N 16°19.767'W	Sweet potato cv. Patagallo	No symptoms	+
CI8	Tenerife	28°34.346'N 16°19.766'W	Sweet potato cv. Patagallo	No symptoms	+
CI9	Tenerife	28°34.341'N 16°19.767'W	Sweet potato cv. Blanca	No symptoms	-
CI10	Tenerife	28°34.343'N 16°19.764'W	Sweet potato cv. Blanca	No symptoms	-
CI11	Tenerife	28°34.345'N 16°19.768'W	Sweet potato cv. Roja de Lanzarote	No symptoms	+
CI12	Tenerife	28°34.205'N 16°19.576'W	Sweet potato cv. Blanca	No symptoms	-
CI13	Tenerife	28°34.208'N 16°19.576'W	Sweet potato cv. Blanca	No symptoms	-
CI14	Tenerife	28°34.066'N 16°19.707'W	*Ipomoea indica*	No symptoms	-
CI15	Tenerife	28°31.824'N 16°23.635'W	Sweet potato	No symptoms	-
CI16	Tenerife	28°31.824'N 16°23.636'W	Sweet potato cv. Yaya	No symptoms	+
CI17	Tenerife	28°31.828'N 16°23.639'W	Sweet potato cv. Boquín	No symptoms	-
CI18	Tenerife	28°31.829'N 16°23.638'W	Sweet potato cv. Yema de huevo	No symptoms	-
CI19	Tenerife	28°31.829'N 16°23.637'W	Sweet potato cv. Yema de huevo	No symptoms	+
CI20	Tenerife	28°31.829'N 16°23.633'W	Sweet potato cv. Maní	No symptoms	+
CI21	Tenerife	28°31.831'N 16°23.631'W	Sweet potato	Wrinkled leaves	-
CI22	Tenerife	28°31.831'N 16°23.629'W	Sweet potato cv. Roja de Lanzarote	No symptoms	+
CI23	Tenerife	28°31.833'N 16°23.628'W	Sweet potato cv. Sierra Morena	No symptoms	+
CI24	Tenerife	28°31.831'N 16°23.627'W	Sweet potato cv. Blanca	No symptoms	+
CI25	Tenerife	28°31.830'N 16°23.626'W	Sweet potato cv. Sierra Morena	No symptoms	-
CI26	Tenerife	28°31.828'N 16°23.626'W	Sweet potato cv. Rajadilla	No symptoms	-
CI27	Tenerife	28°32.093'N 16°23.677'W	*Ipomoea indica*	No symptoms	-
CI28	Tenerife	28°34.379'N 16°11.234'W	Sweet potato cv. Amarilla	No symptoms	-
CI29	Tenerife	28°34.380'N 16°11.234'W	Sweet potato cv. Roja de Lanzarote	No symptoms	+
CI30	Tenerife	28°34.377'N 16°11.234'W	Sweet potato cv. Conejera	No symptoms	+
CI31	Tenerife	28°34.383'N 16°11.228'W	Sweet potato	No symptoms	-
CI32	Tenerife	28°34.384'N 16°11.228'W	Sweet potato cv. Amarilla	No symptoms	-
CI33	Tenerife	28°34.369'N 16°11.228'W	Sweet potato cv. Blanca de Lanzarote	No symptoms	+
CI34	Tenerife	28°34.372'N 16°11.228'W	*Ipomoea indica*	No symptoms	-
CI35	Tenerife	28°34.374'N 16°11.245'W	Sweet potato cv. Francesilla	No symptoms	-
CI36	Tenerife	28°34.426'N 16°11.276'W	Sweet potato cv. Conejera	No symptoms	+
CI37	Tenerife	28°34.425'N 16°11.274'W	Sweet potato cv. Rápida	No symptoms	+
CI38	Tenerife	28°34.418'N 16°11.272'W	Sweet potato cv. De Jardín	No symptoms	+
CI39	Tenerife	28°34.418'N 16°11.272'W	Sweet potato cv. Rajadilla	No symptoms	-
CI40	Tenerife	28°34.413'N 16°11.285'W	*Ipomoea indica*	No symptoms	-
CI41	Tenerife	28°28.555'N 16°19.996'W	*Ipomoea indica*	Wrinkled and yellow leaves	-
CI42	Tenerife	28°28.555'N 16°19.973'W	*Ipomoea indica*	Wrinkled and yellow leaves	-
CI43	Tenerife	28°28.555'N 16°19.965'W	*Ipomoea indica*	Wrinkled and yellow leaves	-
CI44	Tenerife	28°28.568'N 16°24.746'W	*Ipomoea indica*	No symptoms	-
CI45	Tenerife	28°28.574'N 16°24.769'W	*Ipomoea indica*	No symptoms	-
CI51	Tenerife	28°22.818'N 16°41.515'W	*Ipomoea indica*	No symptoms	-
CI52	Gran Canaria	28°00.180'N 15°24.427'W	Sweet potato cv. Amarilla nueva	No symptoms	+
CI53	Gran Canaria	28°00.181'N 15°24.427'W	Sweet potato cv. Amarilla nueva	No symptoms	+
CI54	Gran Canaria	28°00.245'N 15°24.478'W	Sweet potato cv. Amarilla	No symptoms	+
CI55	Gran Canaria	28°00.253'N 15°24.473'W	Sweet potato cv. Amarilla	No symptoms	+
CI56	Gran Canaria	28°00.276'N 15°24.415'W	Sweet potato cv. Amarilla	No symptoms	+
CI57	Gran Canaria	28°00.278'N 15°24.382'W	Sweet potato cv. Blanca	No symptoms	+
CI58	Gran Canaria	28°00.286'N 15°24.387'W	Sweet potato cv. Blanca	No symptoms	+
CI59	Gran Canaria	28°00.257'N 15°24.288'W	*Ipomoea indica*	No symptoms	-
CI60	Gran Canaria	28°01.718'N 15°26.723'W	Sweet potato cv. Bali	No symptoms	+
CI61	Gran Canaria	28°01.718'N 15°26.723'W	Sweet potato cv. Bali	No symptoms	+

CI62	Gran Canaria	28°01.755'N 15°26.708'W	Sweet potato cv. Lanzarote	No symptoms	-
CI63	Gran Canaria	28°01.756'N 15°26.706'W	Sweet potato cv. Lanzarote	Wrinkled leaves	-
CI64	Gran Canaria	28°01.773'N 15°26.700'W	Sweet potato	Variegated leaves	-
CI65	Gran Canaria	28°01.775'N 15°26.699'W	Sweet potato	Variegated leaves	-
CI66	Gran Canaria	28°07.881'N 15°38.549'W	Sweet potato cv. Yema de huevo	No symptoms	+
CI67	Gran Canaria	28°08.280'N 15°38.156'W	Sweet potato cv. Yema de huevo	No symptoms	+
CI68	Gran Canaria	28°08.277'N 15°38.157'W	Sweet potato cv. Yema de huevo	No symptoms	+
CI69	Gran Canaria	28°08.255'N 15°38.268'W	Sweet potato	No symptoms	-
CI70	Gran Canaria	28°08.314'N 15°38.312'W	Sweet potato	No symptoms	-
CI71	Gran Canaria	28°08.315'N 15°38.311'W	Sweet potato	No symptoms	-
CI72	Gran Canaria	28°08.315'N 15°38.313'W	Sweet potato	No symptoms	-
CI73	Gran Canaria	28°06.974'N 15°34.550'W	Sweet potato cv. Amarilla	No symptoms	+
CI74	Gran Canaria	28°06.986'N 15°34.556'W	Sweet potato cv. Amarilla	Yellow leaves	+
CI76	Gran Canaria	28°02.584'N 15°28.636'W	*Ipomoea indica*	Yellow leaves	-
CI77	Gran Canaria	28°02.606'N 15°28.631'W	*Ipomoea indica*	Wrinkled and yellow leaves	-
CI78	Gran Canaria	28°01.843'N 15°29.758'W	*Ipomoea indica*	No symptoms	-
CI79	Gran Canaria	28°01.535'N 15°30.281'W	*Ipomoea indica*	No symptoms	-
CI80	Gran Canaria	28°01.541'N 15°30.278'W	*Ipomoea indica*	Wrinkled and yellow leaves	-
CI81	Gran Canaria	28°01.335'N 15°30.663'W	*Ipomoea indica*	No symptoms	-
CI82	Gran Canaria	28°03.484'N 15°34.002'W	*Ipomoea indica*	Wrinkled and yellow leaves	-
CI83	Gran Canaria	28°03.785'N 15°33.182'W	*Ipomoea indica*	Wrinkled leaves and yellow veins	-
CI84	Gran Canaria	28°05.608'N 15°29.993'W	*Ipomoea indica*	Wrinkled and yellow leaves	-
CI85	Gran Canaria	28°05.599'N 15°30.004'W	*Ipomoea indica*	Wrinkled and yellow leaves	-
CI86	Gran Canaria	27°54.447'N 15°30.588'W	*Ipomoea indica*	No symptoms	-
B1	Málaga	36°45.061'N 04°03.388'W	Sweet potato cv. Beauregard	No symptoms	+
B2	Málaga	36°45.063'N 04°03.388'W	Sweet potato cv. Beauregard	No symptoms	+
B3	Málaga	36°45.069'N 04°03.387'W	Sweet potato cv. Beauregard	No symptoms	+
B4	Málaga	36°45.072'N 04°03.389'W	Sweet potato cv. Beauregard	No symptoms	+
B5	Málaga	36°45.070'N 04°03.373'W	Sweet potato cv. Beauregard	No symptoms	+
B6	Málaga	36°45.044'N 04°03.388'W	Sweet potato	No symptoms	-
B7	Málaga	36°45.040'N 04°03.386'W	Sweet potato	No symptoms	-
B8	Málaga	36°45.042'N 04°03.380'W	Sweet potato	No symptoms	-
B9	Málaga	36°45.051'N 04°03.386'W	Sweet potato	No symptoms	-
B10	Málaga	36°45.052'N 04°03.380'W	Sweet potato	No symptoms	-
B11	Málaga	36°45.047'N 04°03.187'W	Sweet potato cv. Morada	No symptoms	-
B12	Málaga	36°45.046'N 04°03.191'W	Sweet potato cv. Morada	No symptoms	-
B13	Málaga	36°45.041'N 04°03.188'W	Sweet potato cv. Morada	No symptoms	-
B14	Málaga	36°45.045'N 04°03.205'W	Sweet potato cv. Morada	No symptoms	+
B15	Málaga	36°45.042'N 04°03.185'W	Sweet potato cv. Morada	No symptoms	-

*Infected and non-infected plants are represented with plus (+) and negative (-) symbols, respectively.
